# MetaDetection: wireless intelligent detection of multiliquids by using space-time-coding metasurface

**DOI:** 10.1093/nsr/nwaf245

**Published:** 2025-06-13

**Authors:** Shi Han Dai, Yan Shi, Yuan Hu, Quan Wei Wu, Hai Yang Tang, Qi Xiang You, Zan Kui Meng, Long Li

**Affiliations:** School of Electronic Engineering, Xidian University, Xi'an 710071, China; School of Electronic Engineering, Xidian University, Xi'an 710071, China; School of Electronic Engineering, Xidian University, Xi'an 710071, China; School of Electronic Engineering, Xidian University, Xi'an 710071, China; School of Electronic Engineering, Xidian University, Xi'an 710071, China; School of Electronic Engineering, Xidian University, Xi'an 710071, China; School of Electronic Engineering, Xidian University, Xi'an 710071, China; School of Electronic Engineering, Xidian University, Xi'an 710071, China

**Keywords:** intelligent sensing system, software-defined radio technology, spatiotemporal coding programmable metasurfaces, orthogonal frequency division multiplexing (OFDM), artificial intelligence

## Abstract

The increasing demand for public safety has created an urgent need for high-performance technologies capable of detecting hazardous liquids with high accuracy, efficiency, and cost-effectiveness. Conventional liquid detection methods often fall short in addressing these requirements due to limitations in precision, operational complexity, and scalability. This study introduces a wireless intelligent system for the detection of suspicious liquids, leveraging advancements in programmable metasurface and software defined radio technologies. By employing a spatiotemporal coding metasurface to transmit orthogonal frequency division multiplexing (OFDM) Wi-Fi signals, the system efficiently manipulates the spectral harmonic distribution of OFDM subcarriers, thereby creating multiple independent detection channels. Artificial intelligence (AI)-based classification algorithms are integrated to extract liquid-specific features from the channel state information (CSI), enabling precise identification of liquid properties. The proposed system exhibits robust immunity to ambient interference, such as interfering signals, temperature fluctuations, and humidity, while achieving near-ideal accuracy in the simultaneous detection and classification of multiple liquids. This innovative approach provides a cost-effective, scalable, and intelligent solution for hazardous substance detection, with transformative potential for security screening and public safety applications.

## Teaser

An intelligent wireless sensing system integrating spatiotemporal coding metasurfaces, universal software radio peripheral, and artificially intelligent algorithms is demonstrated, enabling simultaneous multi-liquid identification with unprecedented discrimination accuracy and interference resistance.

## INTRODUCTION

Research on target identification technology has garnered significant attention across various fields, with hazardous liquid detection emerging as a critical area of focus due to its implications for public safety, security, and environmental protection [1,2]. Illegal liquids, often transported in sealed vessels, pose significant risks, including accidental spills, leaks, or intentional misuse, making their detection a priority in routine security checks. While manual inspection represents the simplest form of liquid identification, it is labor-intensive and incapable of determining the specific composition of liquids. Sensor-based technologies [3,4], which rely on direct contact with liquids to measure physical and chemical properties such as pH levels, conductivity, and refractive index, have been widely adopted. However, direct contact introduces risks of liquid contamination and sensor corrosion, limiting their practicality.

In contrast, non-contact liquid identification methods leverage advanced sensing technologies [5–9] and data analysis techniques [10,11] to acquire accurate and reliable liquid characterization without the drawbacks of contamination, sensor fouling, or corrosion. Among these methods, Raman spectroscopy stands out as a widely used approach [12,13]. By analyzing the inelastic scattering of light, Raman spectroscopy provides detailed molecular information about a liquid's composition, offering high specificity and resolution for identifying a wide range of liquids. Nevertheless, high-quality Raman spectrometers are costly, and their performance can be compromised by environmental factors such as temperature fluctuations and ambient light.

Microwave detection, on the other hand, offers a flexible and versatile alternative for liquid analysis, owing to its insensitivity to environmental conditions. Radio frequency (RF)-based sensing, in particular, exploits the interaction between RF signals and liquids with varying dielectric properties, resulting in changes to channel state information (CSI) that enable remote liquid identification [14–17]. In RF sensing systems, antennas play a critical role in transmitting and receiving electromagnetic waves. Increasing the number of antennas enhances channel capacity and facilitates liquid identification in both line-of-sight (LOS) and non-line-of-sight (NLOS) scenarios [18,19]. However, this approach introduces challenges such as complex hardware systems, large physical footprints, and intricate signal processing algorithms.

Metasurfaces, as two-dimensional (2D) artificially designed structures, have revolutionized the manipulation of electromagnetic waves [20]. By tailoring subwavelength unit cell topologies and functional array arrangements, metasurfaces exhibit extraordinary capabilities and have found applications in wireless communication and sensing [21–26], holographic imaging [27], and radar cross-section (RCS) reduction [28,29], among others. The advent of digital-coding metasurfaces and programmable metasurfaces has further enabled real-time control of electromagnetic characteristics through simplified hardware configurations [30]. By dynamically switching tunable components and materials embedded in metasurface unit cells, these advanced structures offer unprecedented flexibility and functionality in electromagnetic wave manipulation [31–34].

In this article, we propose a digital programmable metasurface-based sensing system for the dynamic identification of multiple liquids. By incorporating a spatiotemporal coding scheme into the programmable metasurface, the proposed method exploits the nonlinear response of the metasurface to generate higher-order harmonic radiation. When illuminated by a set of orthogonal frequency division multiplexing (OFDM) subcarriers, the metasurface effectively modulates the incident waves to produce multiple harmonic frequencies, thereby establishing several independent identification channels. Through optimized spatiotemporal coding patterns, the system directs these harmonic beams precisely toward target liquids located at different spatial orientations. The resulting CSI, containing distinct liquid signatures, is subsequently processed using advanced artificial intelligence (AI) algorithms for accurate liquid classification. Compared with single tone signals, the broadband characteristics of OFDM signals, comprising multiple subcarriers at different frequencies, enable comprehensive frequency-dependent CSI acquisition. This rich spectral data provides enhanced discrimination capability for accurate liquid characterization. The versatile and cost-effective technology enables real-time and precise classification of multiple liquids, offering a practical and efficient solution for the detection of hazardous substances.

## RESULTS

### System architecture for multi-liquid identification

**Figure 1.**
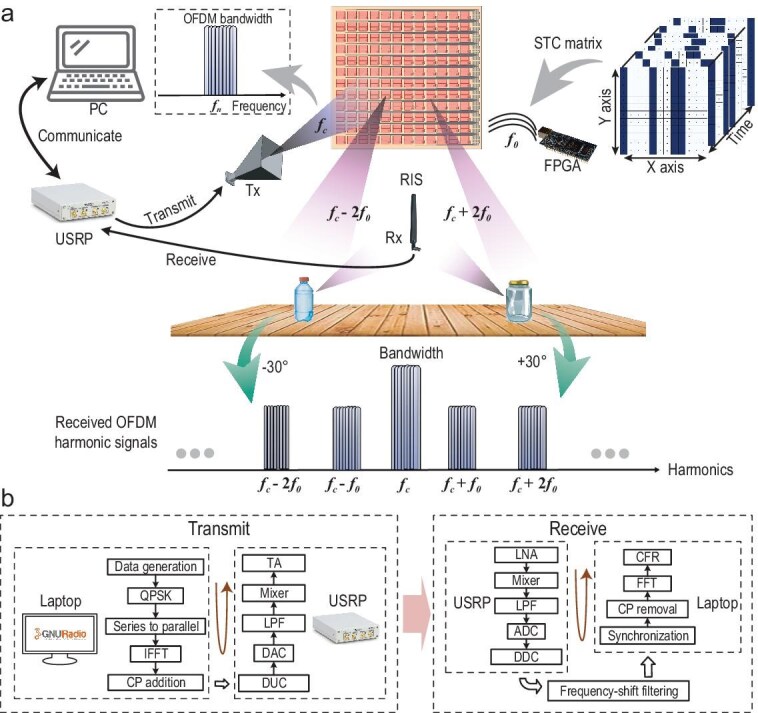
Conceptual framework of a multi-liquid identification system based on spatiotemporal coding metasurface. (a) The system, which is composed of a metasurface illuminated by a feeding horn and controlled by a control circuit, a receiving antenna, a PC, a USRP, and multiple liquids to be detected, transmits the OFDM harmonic signals to illuminate the liquids under test. (b) Flowchart of data processing at transmitter and receiver.

The conceptual framework of the proposed multi-liquid identification system is depicted in Fig. 1. The system comprises a spatiotemporal coding metasurface illuminated by a feeding pyramidal horn antenna, a receiving monopole antenna, a personal computer (PC), and a universal software radio peripheral (USRP) device. The metasurface is dynamically controlled by a field-programmable gate array (FPGA)-based circuit, while the USRP device interfaces with the feeding horn, the receiving antenna, and the PC. An OFDM signal is generated on the PC, modulated by the USRP, and transmitted via the feeding horn antenna. The metasurface, governed by the FPGA control circuit, transforms the incident signal into multiple radiation beams, each characterized by distinct frequencies and propagating along predefined directions. These beams individually illuminate the liquids positioned along their respective paths. The signals scattered by the multiple liquids are captured by the monopole antenna and subsequently transmitted to the PC for analysis. Notably, AI classification algorithms are implemented on the PC to accurately identify and distinguish the different liquids.

To elucidate the operating mechanism of the liquid identification, we consider an ideal model consisting of an infinite large slab with thickness *d*, relative permittivity *ε_r_*, and relative permeability *μ_r_*, embedded in the air. Assuming a unit-amplitude plane wave propagating along the +*z* direction *e*^−^*^jkz^* normally illuminates the slab, in which *k* = 2π*f*/*c* represents wave number, *c* is light speed in free space, and *f* is frequency of the incident wave. Here the time-harmonic convention *e^jωt^* is employed. According to the plane wave propagation theory [35], the reflection coefficient *S*_11_ can be derived as


(1) \begin{eqnarray*}
{S}_{11} = \frac{{ - \frac{j}{2}\left( {\frac{1}{z} - z} \right)\sin (nkd)}}{{\cos (nkd) + \frac{j}{2}\left( {\frac{1}{z} + z} \right)\sin (nkd)}},
\end{eqnarray*}

in which $z = \sqrt {{{{\mu }_r} \mathord{/ {\vphantom {{{\mu }_r} {{\varepsilon }_r}}}} {{\varepsilon }_r}}} $ denotes normalized impedance and $n = \sqrt {{\mu }_r{\varepsilon }_r} $ represents the refractive index. Eq. (1) demonstrates that the reflection wave amplitude is strongly dependent on both the slab's material parameters and the incident wave frequency. Consequently, liquid identification can be achieved by analyzing the scattering wave amplitudes generated by the OFDM signals interacting with the liquid samples.

### OFDM signal generation

**Figure 2.**
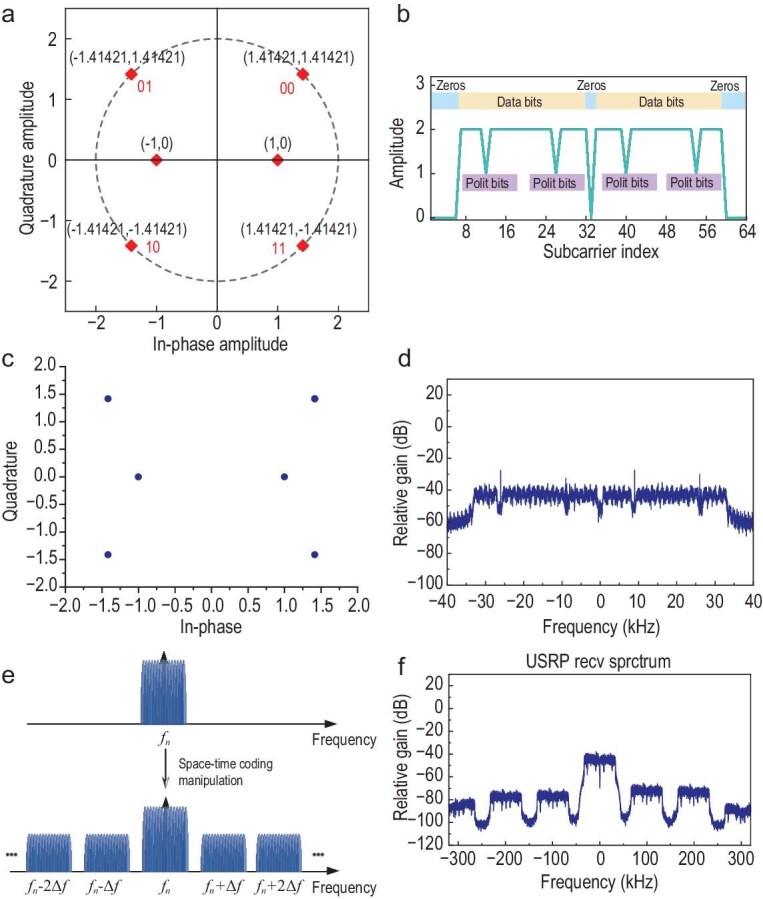
Generation and transmission of OFDM subcarriers. (a) Simulated QPSK + BPSK mapping for bit data modulation. (b) Simulated OFDM subcarriers consisting of null subcarriers, polit subcarriers, and data subcarriers. (c) Measured QPSK + BPSK constellation mapping. (d) Measured OFDM subcarriers. (e) The OFDM subcarriers modulated at different harmonics by using a spatiotemporal coding metasurface. (f) Measured OFDM subcarriers at the USRP receiver.

The OFDM signal generation process, as illustrated in Fig. 1b, was implemented using GNU radio, an open-source software platform, on a Linux-based PC equipped with an Intel i7-8700 CPU and 16 GB RAM. In this system, the digital signal is transformed into a sequence of bits generated at consecutive sample intervals, followed by modulation using a quadrature phase shift keying (QPSK) scheme, as illustrated in Fig. 2a. The modulated symbols are divided into smaller parallel subcarrier streams, which include null subcarriers, polit subcarriers, and data subcarriers, as shown in Fig. 2b. Null subcarriers inserted at the edges of the passband are used to mitigate inter-carrier interference (ICI), eliminating the need for highly efficient bandpass filters or frequency guard bands. In addition to the null carriers at the passband edge, the DC subcarrier, whose phase and magnitude are susceptible to center frequency effects, is intentionally set to zero. Pilot subcarriers are employed to establish a reference for subsequent signal estimation and synchronization processes. Specifically, the data on the pilot subcarriers is modulated utilizing binary phase shift keying (BPSK), as shown in Fig. 2a. Using the inverse fast Fourier transform (IFFT), the multi-carrier OFDM samples in the frequency domain are converted into orthogonal time-domain samples. To prevent inter-symbol interference (ISI), the last quarter of the time-domain samples is copied and inserted at the beginning of the OFDM samples as a cyclic prefix (CP).

We initially generated 108 random data points, which were subsequently mapped into QPSK modulation symbols. The OFDM transmission scheme was configured with 64 subcarriers, comprising 48 data subcarriers, 4 pilot subcarriers, and 12 null subcarriers. The QPSK modulation symbols were allocated to data subcarriers to generate parallel data streams, forming OFDM data frames. Each frame then underwent a 64-point IFFT operation to convert the frequency-domain signal into its time-domain representation. Figure 2c and d depict the measured QPSK constellation mapping and OFDM subcarriers, which are in good agreement with the simulated ones.

The transmission of the modulated OFDM subcarriers is flexibly controlled by configuring parameters such as the sampling rate, master clock, and carrier frequency. Through the universal serial bus (USB) interface connecting the PC and the USRP, the OFDM signal on the USRP is interpolated at the master clock rate using digital-up conversion (DUC), while an analog signal is generated via a digital-to-analog conversion (DAC) circuit. The analog signal, comprising *P* OFDM subcarriers, undergoes filtering via a low-pass filter (LPF) and mixing through a mixer prior to being transmitted via the feeding horn antenna, and can be expressed as


(2) \begin{eqnarray*}
x(t) = \sum\limits_{n = - {P \mathord{\left/ {\vphantom {P 2}} \right. } 2}}^{{P \mathord{\left/ {\vphantom {P 2}} \right. } 2}} {{A}_n{e}^{j2\pi {f}_nt}},
\end{eqnarray*}

where *A_n_* and *f_n_* are amplitude and frequency of the *n*th subcarrier, respectively.

### Design of the programmable metasurface

The programmable metasurface enables dynamic manipulation of electromagnetic waves through real-time, FPGA-controlled unit cells. Assume that the metasurface is composed of *M × N* unit cells, with the periodicities of *d_x_* and *d_y_* along *x* and *y* directions, respectively. The time domain reflective coefficient of (*u, v*)th unit cell is Г*^uv^*(*t*). As the OFDM subcarriers illuminate the programmable metasurface, the scattering field of the metasurface in frequency domain *E*(*θ, φ*) can be obtained by superposing the radiation from all unit cells as


(3) \begin{eqnarray*}
\begin{array}{@{}l@{}} E(\theta ,\varphi ) = \sum\limits_{n = - {P \mathord{\left/ {\vphantom {P 2}} \right. } 2}}^{{P \mathord{\left/ {\vphantom {P 2}} \right. } 2}} {\sum\limits_{u = 1}^M {\sum\limits_{v = 1}^N {{A}_n \cdot } } } \left\{ {{E}^{uv}(\theta ,\varphi ,2\pi f) } \right.\\
\quad\quad\quad\quad \quad \otimes\, {\mathrm{\Gamma}}^{uv}(f) \\
{\left. \cdot {e}^{j{{(2\pi f} \mathord{\left/ {\vphantom {{(2\pi f} {c) \cdot }}} \right. } {c) \cdot }}[ (u - 0.5){d}_x\sin \theta \cos \varphi + (v - 0.5){d}_y \sin \theta \sin \varphi ]} \right\}}_{f = {f}_n}, \end{array}\!\!\!\!\! \nonumber\\
\end{eqnarray*}

in which *θ* and *φ* are elevation and azimuth angles, respectively. The frequency-domain radiation pattern of (*u, v*)th unit cell is *E^uv^*(*θ, φ*, 2*πf*), the frequency domain reflective coefficient Г*^uv^*(*f*) is obtained by performing Fourier transform of Г*^uv^*(*t*), and the symbol $\otimes $ represents the convolution operation. Assuming that the reflection coefficient is periodically manipulated, it can be represented as a composition of *L* pulse waveforms within a single period *T*, i.e.


(4) \begin{eqnarray*}
{{\mathrm{\Gamma }}}^{uv}(t) = \sum\limits_{l = 0}^{L - 1} {{{\mathrm{\Gamma }}}^{uv,l}H(t - l\Delta T)},
\end{eqnarray*}

where


(5) \begin{eqnarray*}
H(t - l\Delta T) = \left\{ \begin{array}{@{}l@{}} 1 \qquad l\Delta T \le t < (l + 1)\Delta T\\ 0 \qquad {\mathrm{ else}} \end{array} \right.,
\end{eqnarray*}

and Г*^uv^,^l^* is the reflection coefficient in *l*th time interval with the interval duration *ΔT* = *T*/*L*. Performing Fourier transform of Eq. (4), Г*^uv^*(*f*) is obtained as


(6) \begin{eqnarray*}
{\Gamma }^{uv}(f) = \sum\limits_{m = - \infty }^{ + \infty } {{r}^{uv,m}\delta (2\pi f - 2\pi m\Delta f)},
\end{eqnarray*}

in which *Δf* = 1/*T* and


(7) \begin{eqnarray*}
\begin{array}{@{}l@{}} {r}^{uv,m} = \displaystyle\frac{1}{L}\displaystyle\frac{{\sin ({{m\pi } \mathord{\left/ {\vphantom {{m\pi } L}} \right. } L})}}{{({{m\pi } \mathord{\left/ {\vphantom {{m\pi } L}} \right. } L})}}{e}^{ - j{{m\pi } \mathord{\left/ {\vphantom {{m\pi } L}} \right. } L}} \\
\quad \quad \quad\quad \displaystyle\cdot\,\sum\limits_{l = 0}^{L - 1} {{\Gamma }^{uv,l}{e}^{ - j{{2ml\pi } \mathord{\left/ {\vphantom {{2ml\pi } L}} \right. } L}}} . \end{array}
\end{eqnarray*}

Thus Eq. (3) can be rewritten as


(8) \begin{eqnarray*}
\begin{array}{@{}l@{}} E(\theta ,\varphi ,\omega ) = \sum\limits_{m = - \infty }^{ + \infty } {\sum\limits_{n = - {P \mathord{\left/ {\vphantom {P 2}} \right. }2}}^{{P \mathord{\left/ {\vphantom {P 2}} \right. }2}} {\sum\limits_{u = 1}^M {\sum\limits_{v = 1}^N {{A}_n{r}^{uv,m} } } } } \\
\quad\quad\quad\quad\quad\quad \cdot\, {E}^{uv}\left[ {\theta ,\varphi ,2\pi ({f}_n - m\Delta f)} \right] \\
\cdot {e}^{j\left[ {{{2\pi ({f}_n - m\Delta f)} \mathord{\left/ {\vphantom {{2\pi ({f}_n - m\Delta f)} c}} \right.} c}} \right] \cdot \left[ {(u - 0.5){d}_x\sin \theta \cos \varphi + (v - 0.5){d}_y\sin \theta \sin \varphi } \right]}. \end{array}\!\!\!\!\! \nonumber\\
\end{eqnarray*}

From Eq. (8), it can be observed that by periodically adjusting the reflection coefficient of each unit cell, the *n*th subcarrier incorporates both the 0th-order and higher-order harmonic components, operating at frequencies *f_n_* and *f_n_* ± *m*Δ*f*, respectively. In this context, multiple groups of OFDM subcarriers modulated at distinct harmonics can be utilized to identify liquids positioned at different locations. Notably, due to the inherent frequency differences among each group of OFDM subcarriers, i.e. *m*Δ*f*, a set of independent channels is established, enabling effective liquid identification, as shown in Fig. 2e and f.

**Figure 3.**
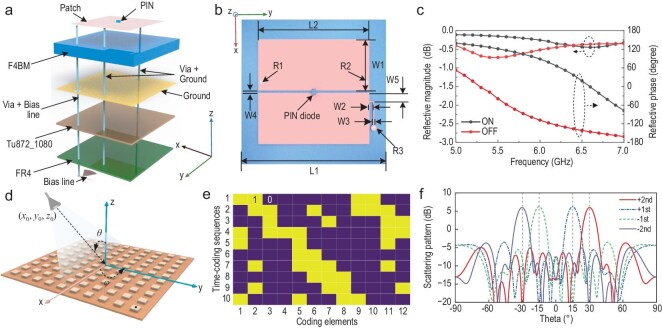
Spatiotemporal coding metasurface. (a) 3D view of the metasurface unit cell. (b) Top view of the unit cell. (c) Reflection magnitude and phase of the unit cell. (d) 3D view of a 12 × 12 metasurface array. (e) 2D spatiotemporal coding sequence. (f) Scattering radiation of different harmonic components. Geometric dimensions of the unit cell: L1 = 25 mm, L2 = 19 mm, W1 = 8.8 mm, W2 = 0.7 mm, W3 = 0.2 mm, W4 = 0.4 mm, W5 = 1.4 mm, R1 = 0.25 mm, R2 = 0.25 mm, R3 = 0.5 mm.

Based on the above spatiotemporal coding theory, a programmable metasurface can dynamically manipulate the characteristics of scattering waves at both fundamental and arbitrary harmonic frequencies through the precise design of spatiotemporal coding matrices. This enables flexible control of multi-beam steering, beamforming, and harmonic energy distribution within a 2D plane. The multi-beam steering technique can be employed to flexibly cover regions of interest, facilitating simultaneous detection of multiple targets. Figure 3 depicts the designed metasurface unit cell along with its corresponding 12 × 12 array configuration. The unit cell consists of an F4BM substrate (with a relative permittivity of 3.5 and a loss tangent of 0.003) featuring a metallic ground, and an FR4 substrate (with a relative permittivity of 4.4 and a loss tangent of 0.026). These two substrates are bonded together using a Tu872_1080 bonding layer (with a relative permittivity of 3.65). On the top surface of the F4BM substrate, two identical square patches are fabricated, with a PIN diode (Skyworks SMP1340-040LF) integrated between them. A metallic via is incorporated to establish a connection between one of the patches and the ground plane. Additionally, a biasing circuit, fabricated on the bottom surface of the FR4 substrate, supplies a DC voltage to the PIN diode through two metallic vias, each connected to one of the patches, respectively. The unit cell is simulated using a finite element solver, with periodic boundary conditions applied to emulate an infinite array. Figure 3c presents the reflection magnitude and phase responses under illumination by an *x*-polarized plane wave. When the PIN diode toggles between the ON and OFF states, the reflection amplitude remains above −0.8 dB across the frequency band of 5.5–6.5 GHz. Simultaneously, the phase difference between the two states achieves 180° ± 20°, meeting the requirements for a 1-bit coding element. The 12 × 12 unit cells are configured into an array and illuminated by a horn antenna positioned at coordinates (0, −82, 357)  mm, with an offset angle θ of 12.94° and a focus-to-diameter (F/D) ratio of 1.07, as shown in Fig. 3d. The time-coding sequence length *L* for each unit cell is set to 10. As each unit cell is independently controlled in both time and space, the resulting metasurface's spatiotemporal coding matrix has a dimension of (12, 12, 10). To simplify the design, an identical spatiotemporal coding scheme is applied to the 12 unit cells in each column along the *x*-direction, reducing the spatiotemporal coding matrix dimension to (12, 10). This 2D coding scheme enables effective beam steering in the yoz plane. Without loss of generality, assume that the beams corresponding to the +1st and −1st order harmonic components are directed at +15° and −15°, respectively, while the beams of the +2nd and −2nd order harmonic components are oriented at +30° and −30°, respectively. To minimize interference between different harmonic components and ensure uniform radiation intensity across all harmonics, a multi-objective optimization algorithm, i.e. binary particle swarm optimization (BPSO) algorithm, is employed to optimize the 2D coding scheme. Figure 3e and f present the optimized 2D coding scheme and the corresponding radiation patterns for different harmonic components, respectively. The desired radiation directions for each harmonic are successfully achieved, with nearly uniform radiation intensity observed across all harmonics. Furthermore, in each designated radiation direction, the corresponding harmonic component exhibits the maximum radiation level, exceeding the levels of other harmonics by ∼20 dB.

### Acquisition and processing of CSI

Multiple groups of OFDM subcarriers are transmitted through the metasurface and subsequently scattered by the various liquids under test. The scattered waves are captured by a monopole antenna. The channel associated with the *m*th harmonics can be expressed as


(9) \begin{eqnarray*}
{{\bf Y}} = {{\bf H}} \cdot {{\bf X}} + {{\bf s}},
\end{eqnarray*}

in which **Y** is the received vector, **X** is the transmitted vector composed of *P* OFDM subcarriers, i.e.


(10) \begin{eqnarray*}
\begin{array}{@{}l@{}} {{\bf X}}[n] = \displaystyle\sum\limits_{u = 1}^M {\sum\limits_{v = 1}^N {{A}_n{r}^{uv,m} } } \\
\quad \quad \quad\quad \cdot\,{E}^{uv}\left[ {\theta ,\varphi ,2\pi ({f}_n - m\Delta f)} \right] \\
\cdot\, {e}^{j\left[ {{{2\pi ({f}_n - m\Delta f)} \mathord{\left/ {\vphantom {{2\pi ({f}_n - m\Delta f)} c}} \right.} c}} \right] \cdot \left[ {(u - 0.5){d}_x\sin \theta \cos \varphi + (v - 0.5){d}_y\sin \theta \sin \varphi } \right]}, \end{array}
\end{eqnarray*}

and **s** is additive Gaussian noise vector. Here **H** is channel frequency response (CFR) matrix including the characteristics of the liquids under test. Due to the orthogonality of the OFDM subcarriers associated with different harmonics, the received subcarriers can be efficiently separated in the frequency domain, facilitating accurate liquid identification.

As shown in Fig. 1b, the received signals are processed by the USRP. After passing through a low-noise amplifier (LNA), a mixer, and a LPF, the signals are amplified and down-converted to baseband. An analog-to-digital converter (ADC) is utilized to digitize the baseband signals, followed by digital signal processing through a digital down-converter (DDC) that executes essential operations including frequency mixing, digital filtering, and signal decimation to facilitate subsequent data extraction and analysis.

**Figure 4.**
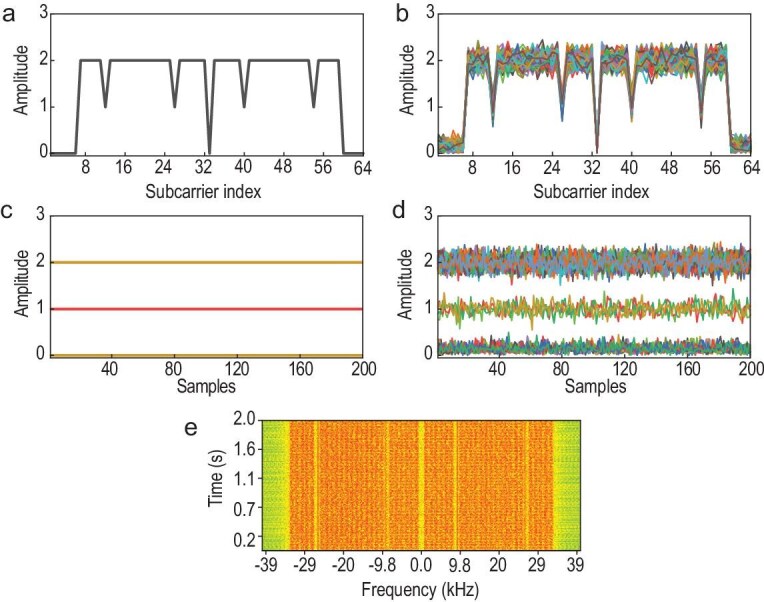
Information derived from the CFR including characteristics of the liquids under test. (a) Ideal CFR. (b) Simulated CFR with additive white Gaussian noise. (c) Amplitude information extracted from the ideal CFR. (d) Simulated amplitude information extracted from the CFR with additive white Gaussian noise. (e) Measured amplitude information extracted from the CFR.

The down-converted signals are transmitted to the PC, where a frequency shift filtering operation is performed using GNU radio to separate the down-converted signals into multiple signals with the same central frequency corresponding to different harmonics. For subsequent demodulation, the CP of each signal frame is initially removed. The Van de Beek synchronization algorithm is then employed, leveraging the CP data to estimate and compensate for both time and frequency offsets. Following this, a fast Fourier transform (FFT) is applied to convert the time-domain OFDM signal into the frequency domain, enabling the extraction of amplitude information from the CFR. Figure 4a–d illustrates the ideal CFR and the CFR in the presence of the additive white Gaussian noise effect at the simulated channel, as well as the corresponding magnitude information. It is evident that, irrespective of noise interference, the magnitude levels of data subcarriers and pilot subcarriers fluctuate around the values of 2 and 1, respectively, while null subcarriers consistently exhibit zero magnitude throughout the transmission. As shown in Fig. 4e, the experimental results confirm excellent agreement with the simulation results after the spatiotemporal coding metasurface-generated multiple harmonic OFDM signal undergoes processing through the USRP receiver.

### AI algorithm for liquid identification

**Figure 5.**
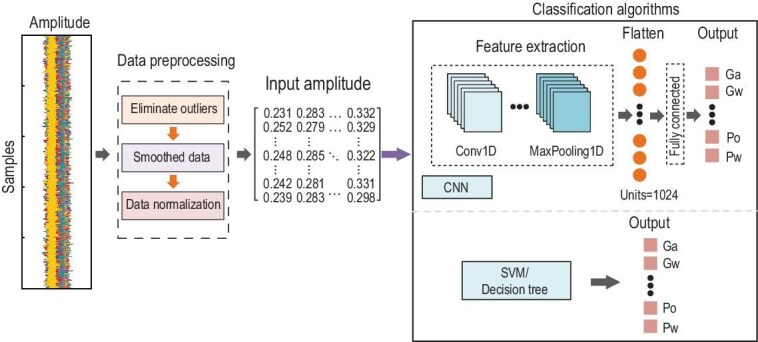
Data processing and AI-driven identification.

The extracted amplitude information of the signal can be utilized for liquid identification through AI classification algorithms. Figure 5 illustrates a two-stage methodology for precise liquid identification. The first stage involves data processing to enhance information quality and relevance, while the second stage employs AI classification algorithms to ensure accurate identification based on the optimized dataset. Notably, while the amplitude, phase and hybrid amplitude-phase information of the received signal could be utilized for liquid identification, practical considerations favor amplitude-based methods. This is because phase information exhibits significantly greater sensitivity to environmental factors, leading to degraded identification accuracy. Consequently, amplitude information emerges as the more robust and suitable choice for reliable liquid identification.

Initially, the amplitude information is organized into a matrix format, with each column representing the data of a specific subcarrier. However, the signal at this stage contains significant noise, as shown in Fig. 5. To effectively eliminate outliers, the syms5 wavelet at level 4 is applied. For processing the detail coefficients, the soft heuristic Stein's unbiased risk estimate (SURE) thresholding technique is employed, with particular attention given to mitigating the influence of scale noise. This approach not only effectively removes outliers but also ensures a clear data transition, enhancing the quality of the signal for subsequent analysis.

To attenuate high-frequency noise components in the acquired signals, data smoothing was implemented via a moving average filter employing discrete convolution processing with a window size of *Q* points, i.e.


(11) \begin{eqnarray*}
{d}_k = \frac{1}{Q}\sum\limits_{t = 0}^{Q - 1} {{b}_{k - t}},
\end{eqnarray*}

where *b_i_* is the unsmoothed original dataset. This linear filtering technique effectively reduces random noise while preserving essential signal trends through its inherent low-pass frequency response characteristics. Here a window size *Q* is set to 100. The smoothed dataset was subsequently normalized to the range of [0, 1] for eliminating scale dependencies and facilitating subsequent analysis through the formulation:


(12) \begin{eqnarray*}
{\bar{d}}_k = \displaystyle\frac{{{d}_k - {d}_{k,{\mathrm{min}}}}}{{{d}_{k,\max } - {d}_{k,{\mathrm{min}}}}},
\end{eqnarray*}

where *d_k_*,_min_ and *d_k_*,_max_ denote the minimal and maximal values of data sample, respectively.

Figure S1 presents the measured results with and without the implementation of smoothing and normalization techniques. The results demonstrate that the application of these preprocessing operations significantly enhances the discriminative characteristics of the dataset, enabling clear differentiation among various liquid properties. Using the normalized dataset, three distinct machine learning algorithms, i.e. support vector machine (SVM), decision tree (DT), and convolution neural network (CNN), were implemented for liquid identification tasks due to their distinct advantages and suitability in target recognition, as shown in Fig. 5. SVM, known for its robustness in high-dimensional spaces, excels in handling non-linear separability through kernel functions, ensuring reliable classification even with complex data distributions. DT provides interpretable decision boundaries and performs well with structured data, making it suitable for preliminary feature analysis. CNN, as a deep learning model, automatically extracts hierarchical features from raw input data, offering superior performance in processing high-dimensional and spatially structured data. By leveraging these three algorithms, the liquid identification performance is comprehensively evaluated.

SVM is a powerful supervised learning algorithm widely used for classification tasks. The fundamental principle of SVM is to find the optimal hyperplane that maximizes the margin between different classes in the feature space. Given a set of training data $\{ {({x}_i,{y}_i)} \}_{i = 1}^n$, where ${x}_i \in {\mathbb{R}}^d$ represents the feature vector and ${y}_i \in \{ { - 1, + 1} \}$ denotes the class label, the goal of SVM is to solve the following optimization problem:


(13) \begin{eqnarray*}
\mathop {\min }\limits_{w,b} \displaystyle\frac{1}{2}{\left\| w \right\|}^2 + C\sum\limits_{i = 1}^n {{\xi }_i},
\end{eqnarray*}

subject to the constraints:


(14) \begin{eqnarray*}
{y}_i(w \cdot {x}_i + b) \ge 1 - {\xi }_i,\quad {\xi }_i \ge 0,\quad \forall i = 1, \cdots ,n.
\end{eqnarray*}

Here, *w* is the normal vector of hyperplane, *b* is the bias term, *ξ_i_* are slack variables that allow for soft-margin classification, and *C* is a regularization parameter that controls the trade-off between maximizing the margin and minimizing the classification error.

The DT is a non-parametric supervised learning algorithm used for classification tasks. It operates by recursively partitioning the feature space into regions, with each partition represented as a node in the tree. The goal is to create a model that predicts the target variable by learning simple decision rules inferred from the data features. The DT algorithm selects the best feature and threshold at each node to split the data into subsets that maximize homogeneity within the resulting subsets. For classification tasks, homogeneity is measured using the metric of Gini impurity, which is defined for a node *t* as:


(15) \begin{eqnarray*}
G(t) = 1 - \sum\limits_{k = 1}^K {p_{\left. k \right|t}^2} ,
\end{eqnarray*}

where ${p}_{ k |t}$ is the proportion of class *k* in node *t*. The splitting process continues until a stopping criterion is met, i.e. the minimum number of samples per leaf is equal to 1.

The CNN employs a hierarchical architecture to automatically learn discriminative features for classification tasks. The network initially extracts low-level features through convolutional layers, where learnable filters perform local operations to capture spatial patterns. Subsequent pooling layers reduce spatial dimensions while preserving essential features. As the network deepens, these features are progressively combined into higher-level representations through multiple convolutional and pooling operations. Finally, fully connected layers integrate these hierarchical features to perform classification, with the softmax function generating probability distributions over target classes. This end-to-end learning framework enables CNN to effectively capture both local and global patterns within input data, making them particularly suitable for classification tasks.

The proposed CNN framework is designed to process sequential input data characterized by 48 distinct features, corresponding to the frequency-domain data subcarriers, with each feature dimension containing preprocessed and normalized values. The model incorporates three sequential 1D convolutional blocks, each followed by MaxPooling1D operations, to extract hierarchical spatiotemporal features. The convolutional layers progressively increase in filter size (128, 256, 512), while maintaining a consistent kernel size of 3. The extracted features are subsequently flattened and fed into a fully connected layer comprising 1024 units with ReLU activation. To mitigate overfitting, dropout regularization is employed during this process. The model is optimized using Adam (learning rate = 0.001) with categorical cross-entropy loss and trained for 15 epochs with a batch size of 64. The architectural details are illustrated in Fig. 5.

The performance of the AI classification algorithm was evaluated using multiple metrics, including recall, precision, *F*1*_score_*, classification accuracy, sensitivity, specificity, and receiver operating characteristic (ROC) curve. These metrics were calculated based on four fundamental outcomes: true negatives (TNs, representing correctly classified negative samples), true positives (TP, indicating correctly classified positive samples), false negatives (FN, denoting positive samples incorrectly classified as negative), and false positives (FP, representing negative samples incorrectly classified as positive). Specifically, recall, equivalent to sensitivity, measures the proportion of TP correctly identified, while precision quantifies the model's ability to avoid FP. *F*1*_score_* harmonizes these two metrics as their harmonic mean, providing a balanced assessment when class distribution is imbalanced. Classification accuracy, though intuitive, can be misleading in skewed datasets, as it equally weights TP and TN. Specificity, on the other hand, focuses solely on the TN rate, complementing sensitivity (recall) in evaluating a classifier's performance across both classes. The ROC curve integrates sensitivity and (1-specificity) across varying thresholds. The area under the ROC curve called AUC quantifies overall discriminative power, where a higher AUC indicates better model performance in distinguishing between classes. While precision-recall curves are preferred for imbalanced data, ROC analysis remains useful for visualizing the trade-off between sensitivity and specificity. Together, these metrics provide a comprehensive evaluation framework: accuracy for overall correctness, precision-recall for positive class reliability, *F*1*_score_* for balance, and ROC-AUC for threshold-independent class separation assessment. The mathematical expressions for these metrics are as follows:


(16) \begin{eqnarray*}
{\mathrm{recall}} = {\mathrm{sensitivity}} = {{TP} / {(TP + FN)}},
\end{eqnarray*}

(17) \begin{eqnarray*}
{\mathrm{accuracy}} = {{(TP + TN)}} / {(TP + TN + FP + FN)},
\end{eqnarray*}

(18) \begin{eqnarray*}
{\mathrm{precision}} = {TP} / {(TP + FP)},
\end{eqnarray*}

(19) \begin{eqnarray*}
F{1}_{\textit{score}} = {2{\mathrm{precision}} \cdot {\mathrm{recall}}} / {({\mathrm{precision}} + {\mathrm{recall}})},
\end{eqnarray*}

(20) \begin{eqnarray*}
{\mathrm{specificity}} = {{TN} / {(TN + FP)}}.
\end{eqnarray*}

### Measured result discussion

**Figure 6.**
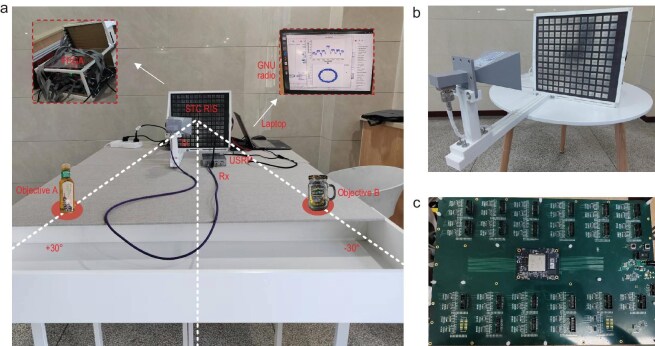
Experimental setup for liquid identification. (a) The whole experimental system. (b) Programmable metasurface array. (c) FPGA-based control circuit.

The experimental setup is illustrated in Fig. 6. The RF signal transmission and reception were implemented using a USRP B210 from Ettus Research Corporation. A standard gain horn antenna with an operating frequency range of 5.3–8.2 GHz was mounted in an offset-feeding configuration in front of the 12 × 12 metasurface array. The control circuit of the metasurface consists of a FPGA (XC7K325TFFG900) integrated with 42 octal bus transceivers (SN74HC245TS). The FPGA serves as the central processing unit that generates control signals, which are subsequently transmitted to the octal bus transceivers. Each transceiver, featuring 6 output channels, facilitates asynchronous bidirectional communication across data buses. This configuration enables precise and independent control of 144 PIN diodes embedded in the metasurface array, ensuring accurate manipulation of the metasurface properties. The transmitter port of the USRP B210 was connected to the feed horn via an RF cable, while a monopole antenna was connected to its receiver port and positioned facing the metasurface array. The optimal placement of the dipole antenna was determined by monitoring the signal spectrum and demodulated constellation diagram in GNU radio, ensuring effective reception and demodulation of higher order harmonics. Two liquid samples were positioned in the horizontal plane along the +30° and −30° directions. This configuration enabled the reception of +2 and −2 order harmonic signals containing the channel response data corresponding to the liquid targets at these angular positions.

The liquid identification system operates at a frequency of 5.8 GHz with an OFDM signal bandwidth of 80 kHz. The designed metasurface array is dynamically controlled by an FPGA module operating at a 50 MHz clock rate. The temporal coding sequence is configured with a modulation period of *T* = 10 μs and a coding sequence length of *L* = 10 within each period, resulting in a minimum harmonic frequency interval of 100 kHz. To establish multiple identification channels utilizing ±1 and ±2 order harmonics, the received OFDM signal requires a bandwidth exceeding 480 kHz. Accordingly, the LPF at the receiver is set to a bandwidth of 640 kHz. The system employs 64-point IFFT and FFT operations at the transmitter and receiver, respectively, to facilitate the transformation of the OFDM signal between the time and frequency domains. For acquisition of the AI-based identification dataset, each experimental sample is repeated 3 times, with 5000 samples collected per trial, which are partitioned into training, test, and validation datasets in an 8:1:1 ratio.

#### Single-target identification

**Figure 7.**
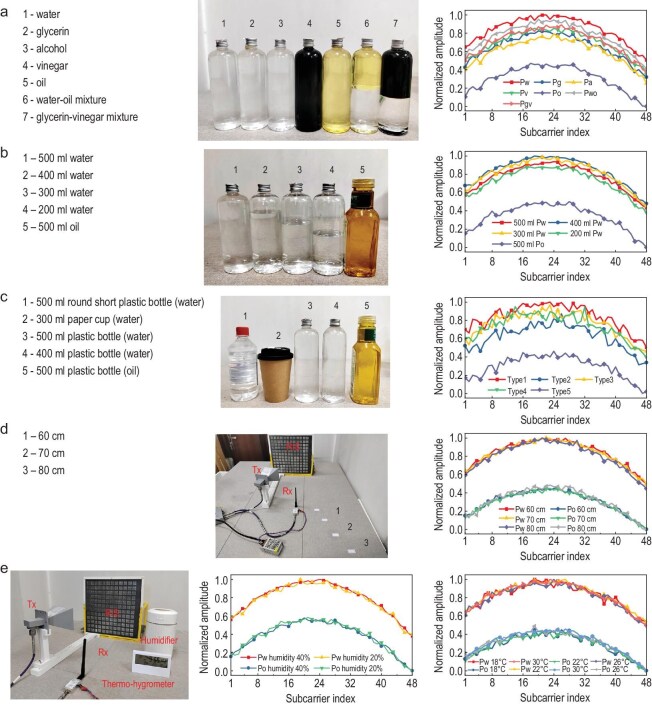
Single-liquid identification performance. (a) Normalized amplitudes of seven liquids with equal volumes in identical plastic bottles. (b) Normalized amplitudes of water at different volumes in the same plastic bottle. (c) Normalized amplitudes of water in the different containers. (d) Normalized amplitudes of water for varying metasurface-to-liquid distances. (e) Normalized amplitudes of water under different ambient temperature and humidity conditions.

We positioned 7 distinct test samples at 30° orientation of the metasurface, corresponding to the +2nd order harmonic location. As shown in Fig. 7a, the sample set consisted of various liquids with identical volumes (500 ml), specifically including water in a plastic bottle (Pw), glycerin in a plastic bottle (Pg), alcohol in a plastic bottle (Pa), vinegar in a plastic bottle (Pv), oil in a plastic bottle (Po), a water-oil mixture in a plastic bottle (Pwo), and a glycerin-vinegar mixture in a plastic bottle (Pgv). This comprehensive selection was designed to evaluate the system's identification capability across different liquid properties. Figure 7a demonstrates the amplitude responses across 48 subcarriers for 7 liquid samples. The results clearly differentiate the characteristic features of various liquid types within identical containers, effectively validating the system's precise liquid identification capability. Figure 7b presents the normalized amplitude responses of the received subcarriers for water samples of varying volumes, all contained in identical plastic bottles. For comparison, the results of oil in a plastic bottle are provided as a reference. The centroid of each liquid sample was carefully positioned at the same elevation as the metasurface center throughout measurements. As the liquid volume decreases, variations in the normalized amplitudes are observed. Figure 7c illustrates the normalized amplitudes of the received subcarriers for water contained in bottles with varying shapes and materials. The observed differences in normalized amplitudes can be attributed to the distinct physical properties of each container type and the volumes of the contained liquid. Figure 7d shows the influence of spacing between the liquid under test and the metasurface on the normalized subcarrier amplitudes. The results reveal that the normalized amplitude profiles for each liquid remain remarkably consistent across different positions, while maintaining a clear distinction between the two liquid types, enabling reliable identification. Figure 7e presents the received signal waveforms under varying ambient temperature and humidity conditions. The ambient temperature is adjusted by air conditioning in the room, while the ambient humidity is controlled by a humidifier positioned 20 cm from the liquid sample. A thermos-hygrometer is used to measure air temperature and humidity. The results reveal that the received signals are insensitive to the ambient temperature and humidity.

To further evaluate the practical applicability of the proposed system, we systematically investigate the influence of noise on liquid identification. Figure S2 demonstrates the impact of interference on liquid identification performance under two scenarios: (1) additive Gaussian noise at −90 dB, and (2) intentional interference signals peaking at −35 dB. In the first case, both OFDM signals and Gaussian noise were transmitted through the USRP. Comparative analysis of Fig. S2b and S2c reveals that while the Gaussian noise introduced by the USRP exceeds the ambient white Gaussian noise by 20 dB, the received harmonic waveforms exhibit remarkable consistency with the reference case in the low-noise condition.

Furthermore, the second case introduces a dedicated interference source (an additional USRP called USPR2) transmitting multiple interfering signals across a 640 kHz bandwidth (Fig. S2a). As evidenced in Fig. S2b and S2d, the interference peaks at −35 dB, surpassed the amplitude of undisturbed received signals. This results in noticeable waveform distortion in the received harmonics, as shown in Fig. S2e. However, subsequent signal processing through the USRP including LNA amplification, mixer down conversion, and LPF filtering, followed by smoothing and normalization, effectively mitigates the interference. Finally, as shown in Fig. S2f, the normalized subcarrier amplitudes remain unaffected, demonstrating the robustness of our identification method against such interference. Notably, the testing environment requires no specialized calibration, as all liquid samples are measured under identical conditions. The proposed system leverages the distinct channel response characteristics of different liquids to OFDM signals for classification, eliminating the need for environmental calibration, which is a significant advantage of the proposed system for practical deployment.

With the normalized amplitudes of the subcarriers, three AI classification algorithms are used to identify the liquids. In the CNN model, the hyperparameters including dataset size, batch size, and learning rate need to be optimized for good classification performance.

**Figure 8.**
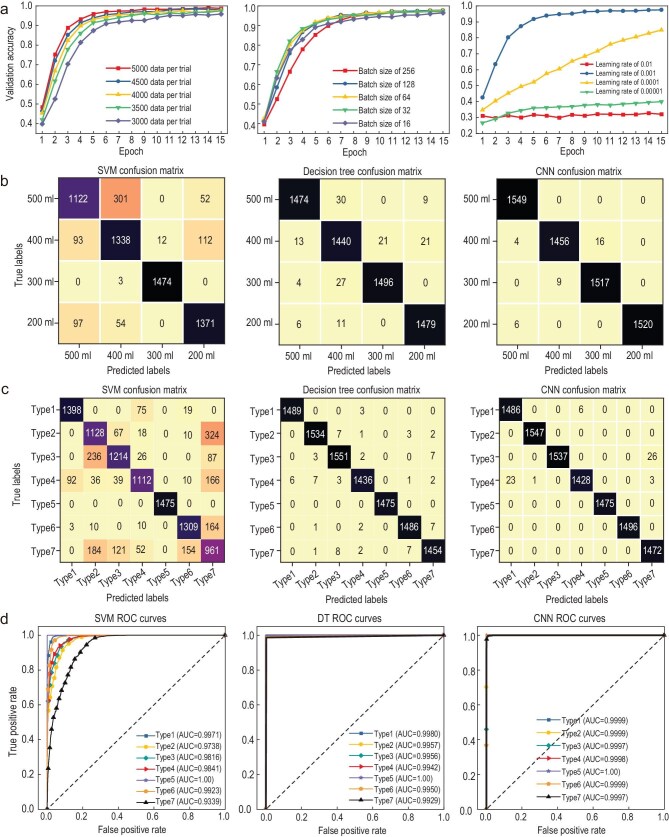
Single-liquid identification performance. (a) Effects of the hyperparameters including dataset size, batch size, and learning rate on the validation accuracy of the CNN model. (b) Confusion matrices of three AI identification algorithms for water with different volumes. (c) Confusion matrices of three AI identification algorithms for seven liquids in the same plastic bottle. (d) ROC curves of three AI identification algorithms for different liquids contained in identical plastic bottles.

In the study, each liquid sample is measured three times to ensure repeatability. Meanwhile, for each measurement trial, the dataset size is systematically varied from 3000 to 5000 samples in increments of 500 to assess its impact on model performance. As shown in Fig. 8a, increasing the dataset size improves both model convergence and validation accuracy. Based on these results, the optimal dataset size of 5000 samples per trial is selected to establish the CNN model. Although larger batch sizes improve validation accuracy, they degrade convergence behavior. Thus, a batch size of 64 is adopted as an optimal balance. Additionally, the highest accuracy is attained at a learning rate of 0.001, establishing it as the preferred choice for model training.

The classification performance of three machine learning algorithms was systematically evaluated using confusion matrices and ROC curve analysis. Figure 8b and c present the confusion matrices for classifying water samples with varying volumes and seven distinct liquid types within identical plastic bottles, respectively. For water volume classification, SVM achieved an identification accuracy of 88%, significantly lower than DT (98%) and CNN (99%). Similarly, in the multi-liquid classification task, SVM attained an 82% accuracy, while both DT and CNN demonstrated superior performance with 99% accuracy. These results highlight the consistent outperformance of DT and CNN over SVM in both experimental scenarios.

Figure 8d quantitatively compares the detection performance of three AI algorithms through their respective ROC curves. All models achieve AUC values between 0.93 and 1.00, which significantly surpasses the random classification baseline (AUC = 0.5), demonstrating a strong discriminative capability. Especially, the DT and CNN models exhibited exceptional performance with consistently high stability (AUC≥0.99) across all test conditions. Its ROC curves consistently approached the ideal top-left corner with smooth trajectories, indicating superior feature extraction capacity.

#### Multi-target identification

First, three distinct target combinations were strategically positioned at both +30° and −30° orientations of the metasurface, corresponding to the +2nd and −2nd order harmonic locations, respectively, as shown in Fig. 6. The target configurations included: (1) oil in a plastic bottle paired with alcohol in a glass bottle, (2) alcohol in a glass bottle paired with another identical sample of alcohol in a glass bottle, and (3) alcohol in a glass bottle paired with oil in a plastic bottle.

**Figure 9.**
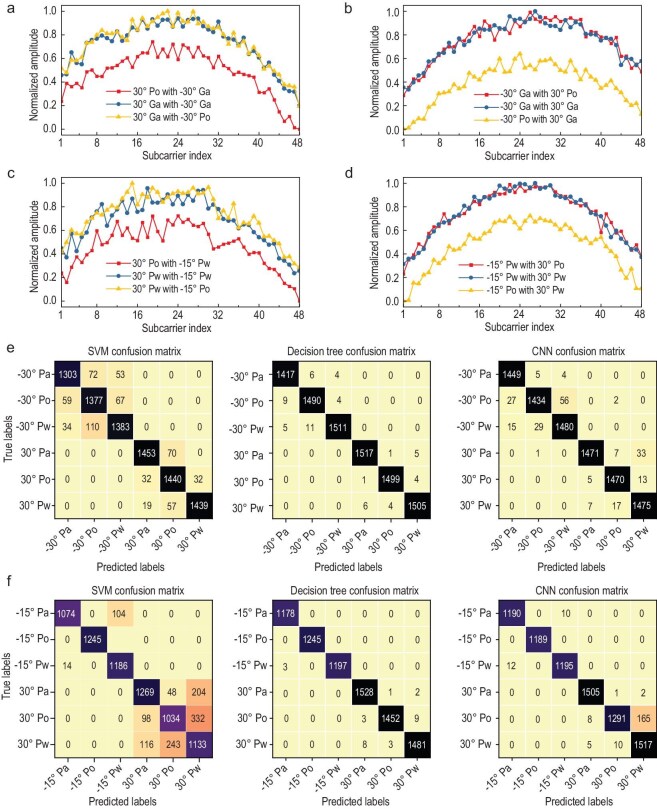
Dual-liquid identification and its performance for symmetrical and asymmetrical positioning. (a) Normalized amplitudes along +30^o^ direction in the symmetrical positioning scenario. (b) Normalized amplitudes along −30^o^ direction in the symmetrical positioning scenario. (c) Normalized amplitudes along +30^o^ direction in the asymmetrical positioning scenario. (d) Normalized amplitudes along −15^o^ direction in the asymmetrical positioning scenario. (e) Confusion matrices of three AI identification algorithms for symmetric positioning. (f) Confusion matrices of three AI identification algorithms for asymmetric positioning.

Figure 9a and b present the normalized amplitude responses along the +30° and −30° directions, respectively. The experimental results demonstrate that the amplitude response of a specific liquid remains consistent regardless of the liquid type present at the other location, effectively establishing two independent identification channels. This characteristic independence is attributed to the metasurface's space-time coding scheme, which has been optimized through the BPSO algorithm to ensure low mutual interference between the two testing directions. Furthermore, the system exhibits distinct amplitude response variations when different liquids are positioned at the same location, thereby enabling reliable and accurate liquid identification. Moreover, it can be also found that the amplitude response of a specific liquid remains remarkably consistent regardless of whether it is positioned along the +30° or −30° direction. This is owing to the metasurface's symmetrical radiation characteristics, which exhibit identical intensity patterns for both +2nd and −2nd order harmonics. These findings collectively validate the system's capability for simultaneous multi-target recognition with minimal mutual interference.

Beyond symmetric positioning, the experimental configuration allows for flexible placement of liquid samples at asymmetric orientations, specifically at +30° and −15° relative to the metasurface, which correspond to the +2nd and −1st order harmonic locations, respectively. Three distinct target combinations were systematically evaluated: (1) oil in a plastic bottle paired with water in a plastic bottle, (2) water in a plastic bottle paired with another identical sample of water in a plastic bottle, and (3) water in a plastic bottle paired with oil in a plastic bottle. Similar to the symmetric positioning scenario, the system successfully establishes two independent identification channels capable of distinguishing amplitude responses of different liquids under asymmetric positioning conditions, as demonstrated in Fig. 9c and d. In addition, comparative analysis reveals similar amplitude response characteristics for a certain liquid in the bottle between the −15° and +30° orientations. This is attributed to the space-time coding optimization of the metasurface which results in similar radiation intensity across the different harmonics.

The confusion matrices in Fig. 9e and f further demonstrate the robust recognition performance of our proposed method under varying configurations. In the symmetric positioning scenario, the SVM model demonstrates a recognition accuracy of 92%, while the DT and CNN models exhibit superior performance, achieving 99% and 98% accuracy, respectively. When evaluating asymmetric positioning conditions, the recognition accuracy measures 86%, 99%, and 97% for the SVM, DT, and CNN models, respectively. Although a marginal decrease in recognition accuracy is observed in asymmetric scenarios compared to symmetric configurations, all models maintain sufficient discriminative capability to effectively distinguish between different liquid types.

**Figure 10.**
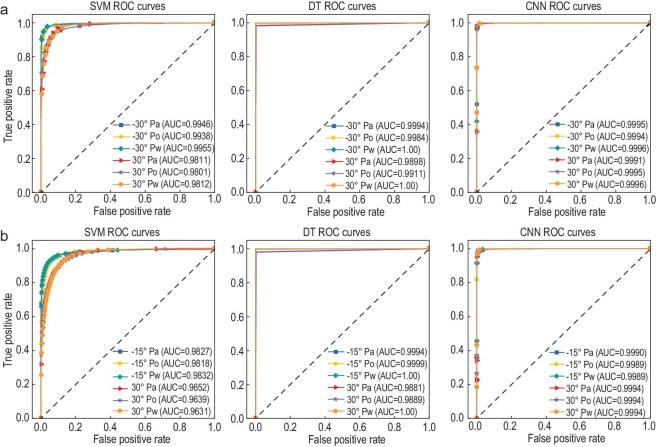
ROC curves of three AI identification algorithms for symmetric and asymmetric positioning. (a) Symmetric positioning. (b) Asymmetric positioning.

Figure 10 presents the ROC curve analysis comparing the performance of three AI identification algorithms in both symmetric and asymmetric positioning scenarios. The evaluation considers three distinct test cases: (1) alcohol positioned at +30° direction versus water at −30°/−15° direction, (2) oil at +30° direction versus water at −30°/−15° direction, and (3) water at +30° direction versus alcohol at −30°/−15° direction. The results demonstrate that the SVM method achieves the AUC values better than 0.98 and 0.96 for symmetric and asymmetric configurations, respectively. In comparison, the DT and CNN algorithms maintain superior performance, with AUC values exceeding 0.98 and 0.99 across all test conditions, respectively. This robust performance highlights the remarkable reliability of the proposed identification system.

#### Target identification in a NLOS scenario

The efficacy of the proposed system in discerning liquids within a NLOS context has been substantiated. Illustrated in Fig. S3, two distinct liquids, namely oil in a plastic bottle and water in a plastic bottle, are positioned at a +30° angle relative to the metasurface. The system's capability was assessed under two conditions: the liquid contained within a cloth bag and the liquid obscured by a wooden board.

The resultant amplitude data, depicted in Fig. S3c, reveal that the normalized magnitude profiles for the liquid remain consistent, irrespective of the presence of an obstruction. This consistency demonstrates the system's robust performance in NLOS environments.

To comprehensively assess the identification performance, Table 1 presents a comparative analysis of five key metrics including precision, recall, *F*1*_score_*, accuracy, and specificity across three AI classification algorithms for three distinct scenarios. The results demonstrate that both DT and CNN consistently outperform SVM across all five metrics in three scenarios. Furthermore, the performance of DT is comparable to that of CNN, indicating their similar efficacy in object identification tasks.

**Table 1.** Comparison of five metrics of the proposed system among different scenarios.

Scenario	Algorithm	Precision	Recall	*F*1*_score_*	Accuracy	Specificity
Single object	SVM	0.90	0.90	0.90	0.90	0.97
	DT	0.97	0.97	0.97	0.97	0.99
	CNN	0.99	0.99	0.99	0.99	0.99
Symmetrically placed	SVM	0.93	0.93	0.93	0.93	0.97
	DT	0.99	0.99	0.99	0.99	0.99
	CNN	0.98	0.98	0.98	0.98	0.99
Asymmetrically placed	SVM	0.87	0.87	0.86	0.86	0.99
	DT	0.99	0.99	0.99	0.99	0.99
	CNN	0.98	0.97	0.97	0.97	0.99

## CONCLUSION

This paper presents an intelligent sensing system based on AI for the simultaneous detection of multiple liquids using wireless signals. A programmable metasurface, controlled in a spatiotemporal manner, is designed to generate multiple independent identification channels through the exploitation of high-order harmonics. By employing OFDM modulation, the subcarriers centered at high-order harmonics exhibit distinct variations when interacting with different liquids in the bottles. To achieve accurate liquid identification, three AI algorithms are integrated into the system. Extensive experiments, including scenarios with single liquids, multiple liquids in symmetric and asymmetric arrangements, and liquids placed in the non-line-of-sight scenario, are conducted to validate the system's effectiveness. The proposed design demonstrates strong immunity to ambient factors and external noise interference, presenting a robust and practical solution for hazardous liquid detection. These characteristics highlight its significant potential for security screening applications.

For real-world deployment, the proposed system requires further consideration of several critical factors. For instance, the current 12 × 12 metasurface prototype employs 144 PIN diodes, consuming 1.224 W in total. Scaling this design to larger arrays would lead to prohibitively high power consumption, underscoring the need for low-power metasurface architectures. Furthermore, the offline-trained AI models used for identification exhibit significant accuracy degradation when encountering new liquid types. Thus, developing online training methods with high computational efficiency represents a critical research direction for practical applications.

## Supplementary Material

nwaf245_Supplemental_File

## Data Availability

All data are available in the main text. Additional data related to this paper may be requested from the corresponding author.
