# Supplementary material for: MetaDetection: wireless intelligent detection of multiliquids by using space-time-coding metasurface
Source: Natl Sci Rev. 2025 Jun 13;12(9):nwaf245. doi: 10.1093/nsr/nwaf245 (PMC12342493; doi:10.1093/nsr/nwaf245)
Supplement: nwaf245_Supplemental_File [file nwaf245_supplemental_file.pdf]

Supplementary Information for

MetaDetection: Wireless intelligent detection of multiliquids by using space-time-coding metasurface

Shi Han Dai[1], Yan Shi[1,*], Yuan Hu[1], Quan Wei Wu[1], Hai Yang Tang[1], Qi Xiang You[1], Zan Kui Meng[1], Long Li[1]

1. *School of Electronic Engineering, Xidian University, Xi'an, Shaanxi 710071, China.*

*Corresponding Emails: shiyan@mail.xidian.edu.cn

The PDF file include:

Fig. 1 to 3

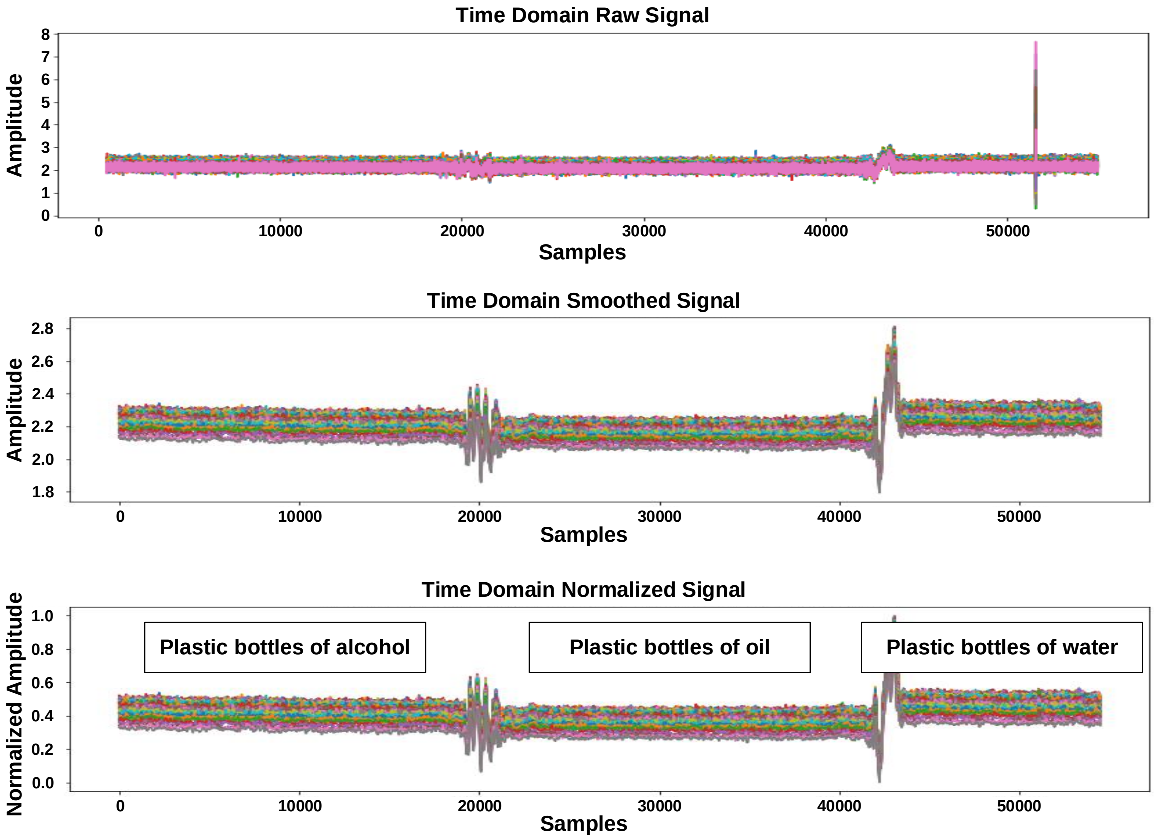

Figure 1. Smoothing and normalization of measured data for AI based liquid identification.

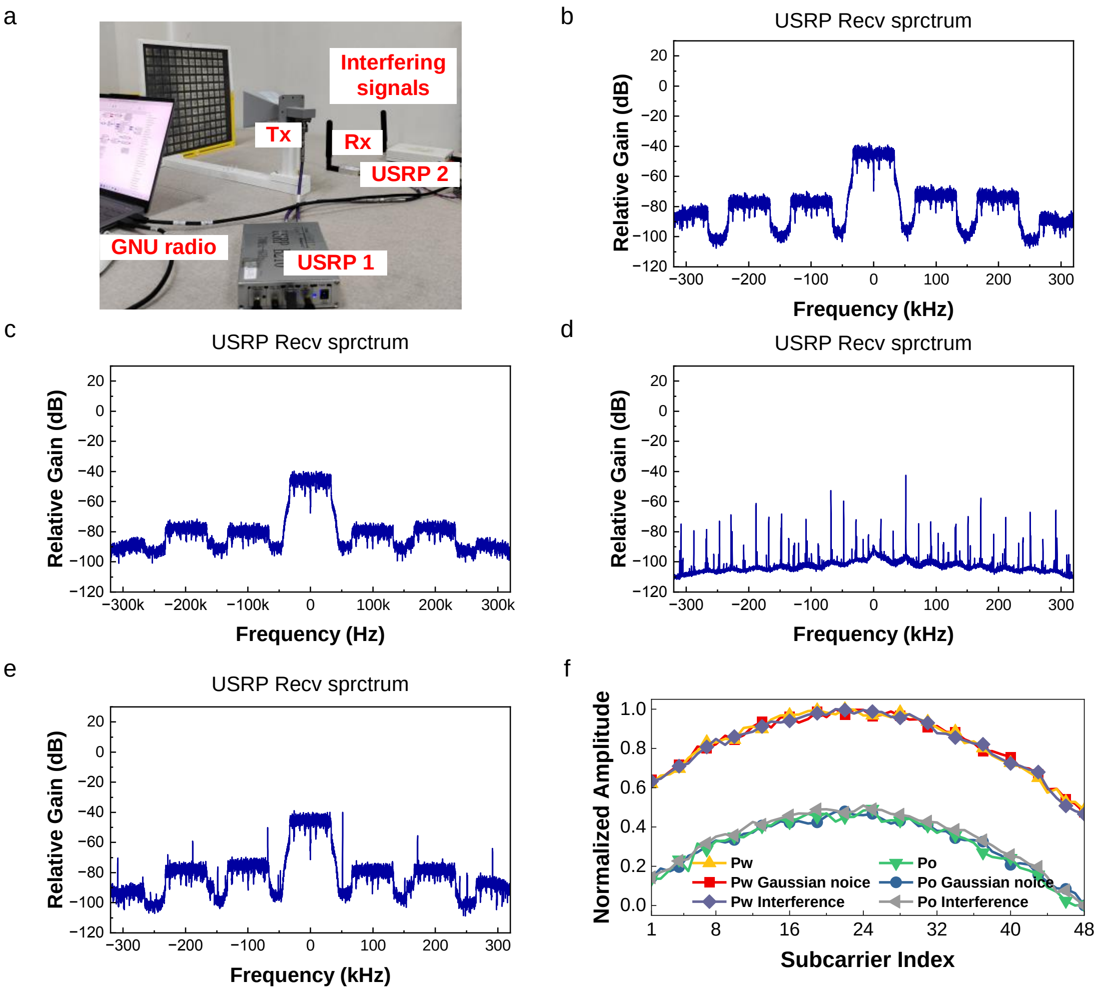

Figure 2 Impact of Gaussian noise and RF interference on liquid identification. (a) Measurement setup with USRP-generated interfering signals. (b) Received harmonic signals with low Gaussian noise. (c) Received harmonic signals with significant Gaussian noise. (d) Interfering signals generated by the USRP. (e) Received harmonic signals with interfering signals. (f) Quantitative interference impact on spectral amplitudes.

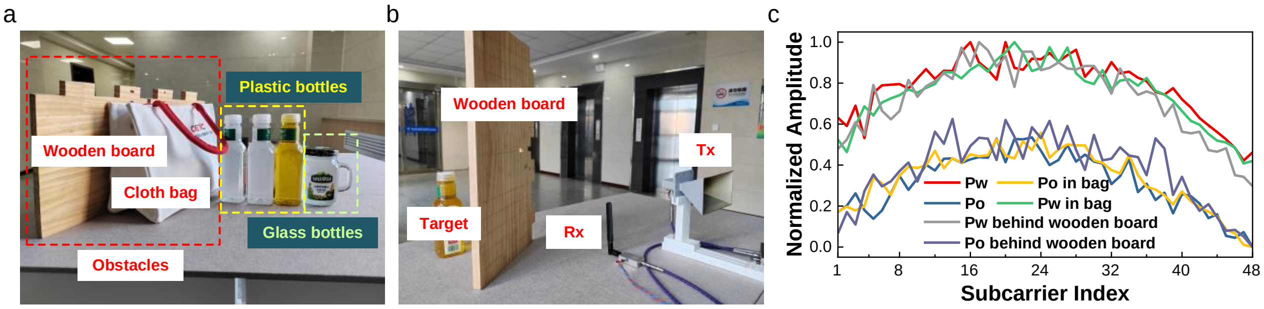

Figure 3 The experiment in NLOS scenario. (a) Water in plastic bottle, oil in plastic bottle, cloth bag, and wooden board. (b) Liquid placed behind the wooden board. (c) Amplitude information along +30° direction.
